# The Elderly Chronic Sick

**Published:** 1953

**Authors:** 


### THE ELDERLY CHRONIC SICK

The Bristol Local Medical Committee is to be congratulated on its rep the treatment and care of the elderly chronic sick of Bristol. The memb the sub-committee who drafted the main report were Dr. H. R. Dutton, fessor A. V. Neale and Dr. W. B. Williams. There are similarities betwee medical problems of the extremes of life, so it was appropriate that Prof Neale should be chairman. The members of the sub-committee acknow their particular indebtedness to Drs. P. C. Joscelyne, W. H. Hayes and Anderson who have held the office of chairman of the L.M.C. in recent and to Dr. W. Hughes and Dr. R. C. Wofinden who helped with the prepa of the report. Appendices on different subjects have been contributed b Hughes, Miss M. E. Burgess and Miss B. L. Robertson.

The report lays stress on the size of the problem and puts forward a p more exact inquiry. It is difficult to separate the problems of the aged from those of the well because the one class shades imperceptibly into the and because there is no definite borderline between those problems whic medical and those which are social. The committee, therefore, found it n sary to go outside its strict terms of reference and to consider, to some e not only the chronic sick but the " frail ambulant ", the acute sick an housing of old people in general.

The problem is an important one and a growing one, and it is to be hoped the excellent lead given by the Local Medical Committee will be followed

